# Elevated Fibroblast growth factor 21 (FGF21) in obese, insulin resistant states is normalised by the synthetic retinoid Fenretinide in mice

**DOI:** 10.1038/srep43782

**Published:** 2017-03-03

**Authors:** Nicola Morrice, George D. Mcilroy, Seshu R. Tammireddy, Jennifer Reekie, Kirsty D. Shearer, Mary K. Doherty, Mirela Delibegović, Phillip D. Whitfield, Nimesh Mody

**Affiliations:** 1 Institute of Medical Sciences, College of Life Sciences and Medicine, University of Aberdeen, Foresterhill Health Campus, Aberdeen, Scotland AB25 2ZD, UK; 2 Centre for Genome Enabled Biology and Medicine, University of Aberdeen, 23 St Machar Drive, Old Aberdeen, Aberdeen, Scotland AB24 3UU, UK; 3 Lipidomics Research Facility, Department of Diabetes and Cardiovascular Science, University of Highlands and Islands, Old Perth Road, Inverness, Scotland IV2 3JH, UK

## Abstract

Fibroblast growth factor 21 (FGF21) has emerged as an important beneficial regulator of glucose and lipid homeostasis but its levels are also abnormally increased in insulin-resistant states in rodents and humans. The synthetic retinoid Fenretinide inhibits obesity and improves glucose homeostasis in mice and has pleotropic effects on cellular pathways. To identify Fenretinide target genes, we performed unbiased RNA-seq analysis in liver from mice fed high-fat diet ± Fenretinide. Strikingly, *Fgf21* was the most downregulated hepatic gene. Fenretinide normalised elevated levels of FGF21 in both high-fat diet-induced obese mice and in genetically obese-diabetic Lepr^*db*^mice. Moreover, Fenretinide-mediated suppression of FGF21 was independent of body weight loss or improved hepatic insulin sensitivity and importantly does not induce unhealthy metabolic complications. In mice which have substantially decreased endogenous retinoic acid biosynthesis, *Fgf21* expression was increased, whereas acute pharmacological retinoid treatment decreased FGF21 levels. The repression of FGF21 levels by Fenretinide occurs by reduced binding of RARα and Pol-II at the *Fgf21* promoter. We therefore establish *Fgf21* as a novel gene target of Fenretinide signalling via a retinoid-dependent mechanism. These results may be of nutritional and therapeutic importance for the treatment of obesity and type-2 diabetes.

Fibroblast growth factor (FGF) 21 is a predominantly liver derived hormone and is being investigated as an anti-obesity/anti-diabetes therapy^1^. FGF21 binds cognate receptors FGFR and β-klotho, which are expressed at high levels in adipose tissue, to mediate its metabolic effects including control of lipid metabolism, body weight and glucose homeostasis^1^,^2^. Hepatic *Fgf21* expression is increased by stimuli such as fasting, protein/amino acid restriction and fructose ingestion via a range of transcription factors, including peroxisome proliferator-activated receptor-alpha (PPARα) and carbohydrate response-element binding protein (ChREBP) 1^3^,^4^,^5^.

Circulating FGF21 levels are also elevated in states of metabolic stress in both rodents and humans and are positively correlated with body mass index and insulin resistance^6^. This has to led the hypothesis that a rise in serum FGF21 levels may be a predictor for metabolic syndrome and type-2 diabetes and that these may be states of relative “FGF21-resistance”^7^. Furthermore, diverse treatments to improve glycaemic control in humans can decrease elevated levels of FGF21 (e.g. with metformin, rosiglitazone, insulin, insulinotropic agents, bariatric surgery, lifestyle modification, fish oil supplements or exercise^8^,^9^,^10^,^11^,^12^,^13^,^14^). Thus, the biological role of FGF21 in obesity and insulin resistance is still not fully explained and further understanding the regulation of FGF21 with the aim of modulating physiological levels maybe key in maximizing its therapeutic potential.

The synthetic retinoid 4-hydroxy(phenyl)retinamide (Fenretinide, FEN) is widely studied as a cancer therapeutic due to its favourable toxicological profile and is currently undergoing phase II clinical trials for treatment of insulin resistance in obese humans with hepatic steatosis^15^,^16^. We have previously shown that FEN prevents obesity and improves insulin sensitivity in both high fat diet (HFD)-fed male and female mice^17^,^18^,^19^. FEN acts via several different mechanisms including induction of retinoid signalling, increased hepatic lipid oxidation and inhibition of the final step of ceramide biosynthesis in multiple tissues to exert its metabolic effects^20^,^21^,^22^. FEN can also directly inhibit gene expression of the satiety hormone leptin and reduce serum levels in mice, but does not have measureable effects on food intake or energy expenditure^17^,^18^. The relationship between FEN and leptin signalling is not fully understood and it is not known whether FEN can modulate *Fgf21* levels.

To further understand the signalling mechanisms of FEN, we performed RNA-seq analysis in liver from FEN-treated mice fed a HFD, which we had previously characterised^18^,^22^. Interestingly, *Fgf21* was the gene most downregulated by FEN treatment. We therefore further tested the potential role of FEN in regulating hepatic *Fgf21* in mouse models of diet and genetically-induced obesity and in wild-type animals.

## Results

### Hepatic *Fgf21* is down regulated in HFD-fed mice receiving Fenretinide treatment

**Figure 1:**
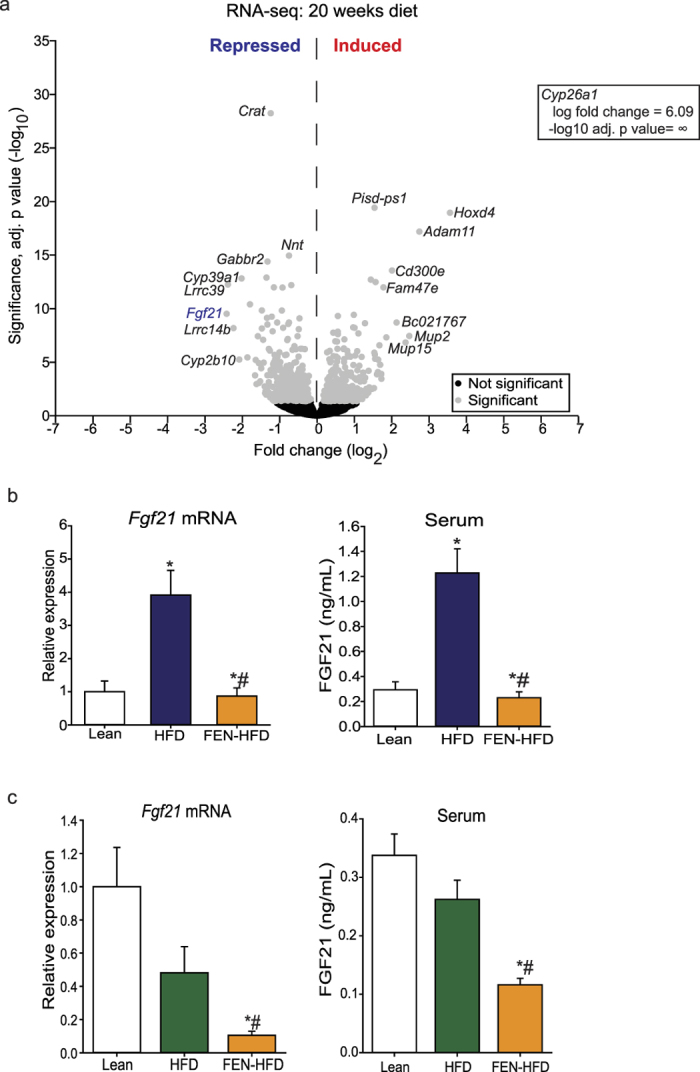
Hepatic *Fgf21* is down regulated in HFD-fed mice receiving Fenretinide treatment. (**a**) Volcano plot of DESeq2 analysis of liver RNA-seq from C57BL/6 mice fed HFD ± FEN for 20 weeks (FEN-HFD; n = 4 mice per group). Grey circles denote significantly changed gene expression (−log_10_ adj. p value < 0.05); black circles denote gene expression unchanged. (**b**) Liver mRNA expression measured by qPCR and serum FGF21 levels in mice fed chow (lean, n = 5), HFD (n = 7) or FEN-HFD (n = 8) for 20 weeks, following 5-hour fast. (**c**) as in (**b**) except diets were fed for 7 days: chow (lean, n = 5), HFD (n = 7) or FEN-HFD (n = 8). Liver gene expression was normalized to *Hprt*. Significant differences were determined by one-way ANOVA followed by *post-hoc* tests. Differences are marked *p < 0.05 vs lean or ^#^p < 0.05 vs HFD.

To identify targets of FEN signalling, RNA-seq analysis was performed in liver from C57BL/6 mice fed a HFD ± 0.04% FEN for 20 weeks. Representative mice (n = 4) from the whole cohort were selected based on the extensive phenotyping performed previously (data in Supplementary Table S1)^18^. Chronic FEN treatment resulted in significant upregulation of 377 genes and 487 were significantly downregulated (Fig. 1a).

The most upregulated genes were classic retinoid targets (cytochrome P450-type enzyme *Cyp26a1*, transcription factor homeobox D4, *Hoxd4* and metalloprotease *Adam11*) which were induced between 6.7 and 68-fold (Fig. 1a). The most significantly downregulated gene was *carnitine acyltransferase (Crat*) whilst, *Fgf21* was the most downregulated gene, repressed 5.4-fold. Both of these genes are established PPARα targets involved in control of lipid metabolism^3^,^23^.

To determine whether FEN can oppose PPARα signalling, we also performed RNA-seq analysis in liver from mice fed a HFD ± FEN for 7 days, which had also been previously characterised (data in Supplementary Table S1)^18^. Acute FEN treatment resulted in significant upregulation of several classic retinoid target genes and *Fgf21* trended to be downregulated. However, FEN treatment did not change the expression of PPARα target genes or target genes of other transcription factors identified as having a role in inducing FGF21, e.g. ChREBP1 (Table 1)^3^,^4^,^24^,^25^.

Gene expression analysis in mice from the whole cohort (n = 7–8), including chow-fed lean animals, confirmed the decrease in *Fgf21* expression in long-term treated mice and FEN also completely normalised elevated serum FGF21 levels (Fig. 1b). In mice receiving the acute diet intervention (HFD ± FEN for 7 days), HFD feeding did not increase *Fgf21*, however FEN decreased FGF21 expression and serum levels more than 50% below normal levels (Fig. 1c). Thus, FEN is able to inhibit *Fgf21* expression both chronically in obese mice and more acutely in lean mice, without inducing parallel changes to known major regulators of hepatic *Fgf21*.

### Fenretinide inhibits *Fgf21* expression and improves glucose homeostasis without preventing weight gain in Lepr^*db*^ genetically obese mice

**Figure 2:**
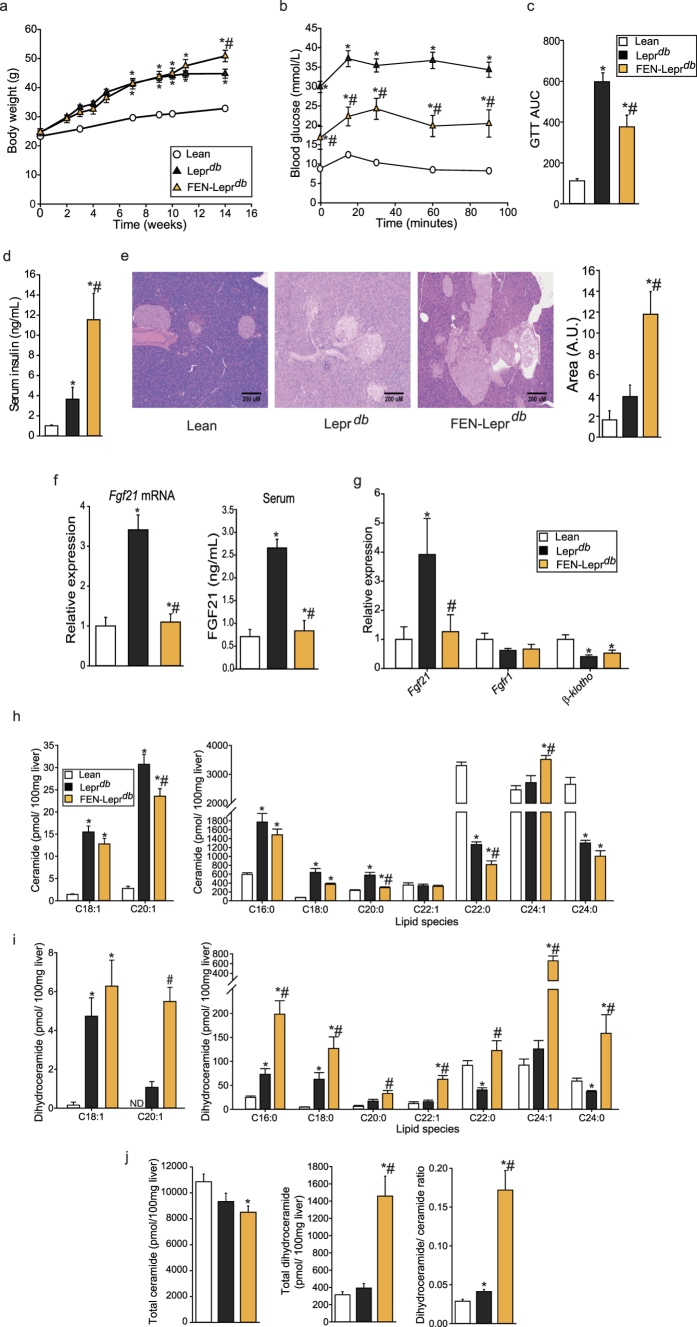
Fenretinide inhibits *Fgf21* expression and improves glucose homeostasis without preventing weight gain in Lepr^*db*^ genetically obese mice. (**a**) Body weight of C57BL/6 lean mice on chow (lean, n = 8; error bars too small to be shown clearly) and C57BL/6 Lepr^*db*^mice on 10% fat (Lepr^*db*^, n = 7) or 10% fat diet +FEN (FEN-Lepr^*db*^, = 7). (**b**) Glucose (1 mg/g body weight) Tolerance Test (GTT) and (**c**) AUC at week 4. (**d**) serum insulin levels at the end of the study, following a 5 h fast. (**e**) Representative photos of haematoxylin and eosin staining of pancreas sections (10x magnification) and quantification of pancreatic islet size (n = 3 per group). (**f**) Hepatic gene expression and serum levels of FGF21. Gene expression was normalised to *Nono*. (**g**) PG-WAT gene expression, normalised to *yWhaz*. Significant differences were determined by one-way ANOVA followed by *post-hoc* tests or student’s t-test for PG-WAT *Fgf21* FEN-Lepr^*db*^ vs Lepr^*db*^ and Lepr^*db*^ vs lean groups and *β-klotho* FEN-Lepr^*db*^ vs Lepr^*db*^ and Lepr^*db*^ vs lean groups. Differences are marked *p < 0.05 vs lean or ^#^p < 0.05 vs Lepr^*db*^. Quantification of ceramide (**h**) and dihydroceramide (**i**) species in liver. (**j**) Total liver levels of ceramide, dihydroceramide and the dihydroceramide/ceramide ratio. Significant differences were determined by one-way ANOVA followed by *post-hoc* tests or student’s t-test for ceramides: C18:0 FEN-Lepr^*db*^ vs Lepr^*db*^ and FEN-Lepr^*db*^ vs lean groups, C20:0 FEN-Lepr^*db*^ vs Lepr^*db*^ and FEN-Lepr^*db*^ vs lean groups, C24:1 FEN-Lepr^*db*^ vs Lepr^*db*^ groups; dihydroceramides: C16:0 FEN-Lepr^*db*^ vs Lepr^*db*^ and Lepr^*db*^ vs lean groups, C18:0 FEN-Lepr^*db*^ vs Lepr^*db*^ and Leprdb vs lean groups, C20:1 FEN-Lepr^*db*^ vs Lepr^*db*^ groups, C20:0 Lepr^*db*^ vs lean groups, C24:0 FEN-Lepr^*db*^ vs lean and Lepr^*db*^vs lean groups. Differences are marked *p < 0.05 vs lean or ^#^p < 0.05 vs Lepr^*db*^.

To investigate the relationship between FEN and leptin signalling and determine effects on FGF21 levels in non-diet-induced obesity, we used the Lepr^*db*^ genetic mouse model. Unlike in HFD mice, FEN did not inhibit rapid weight gain in Lepr^*db*^ mice fed a 10% fat diet (Fig. 2a). FEN did decrease fasting hyperglycaemia and markedly attenuated glucose intolerance in Lepr^*db*^ mice (Fig. 2b,c), in association with increased serum insulin (Fig. 2d) and increased pancreatic islet mass (Fig. 2e). Thus, FEN can improve glucose homeostasis independent of leptin signalling.

Hepatic expression and circulating levels of FGF21 were elevated in Lepr^*db*^ mice but were completely normalised by FEN treatment (Fig. 2f). FEN also suppressed the elevation of *Fgf21* in perigonadal white adipose tissue (PG-WAT) but had no effect on the downregulation of *Fgfr1* or *β-klotho* (Fig. 2g). Several PG-WAT metabolic genes (e.g. *Pparγ* and *Glut4*) were markedly downregulated in Lepr^*db*^ mice. FEN co-ordinately increased the expression of these genes to levels similar to those measured in lean mice and also normalised the elevated expression of PG-WAT *Rbp4* (Table 2).

FEN strongly increased hepatic expression of classic retinoid target genes, as expected (Table 2). FEN had no effect on many genes, circulating factors and metabolites known to have a key role in the control of appetite, adiposity, gluconeogenesis, macrophage infiltration or inflammation (Table 2).

Previous work has shown that FEN treatment results in alterations in ceramide and dihydroceramide levels in HFD obese mice, in association with improvements in glucose homeostasis and insulin sensitivity^20^,^22^. To determine whether FEN could have the same effect in genetically-obese mice, lipid analysis was performed in liver tissue from Lepr^*db*^ mice. A number of ceramide species elevated in Lepr^*db*^ mice were partially decreased by FEN treatment, including C18:0, C20:0 and C22:0 (Fig. 2h). With regards to the immediate precursor to ceramide in its biosynthetic pathway, FEN treatment also resulted in an increase in dihydroceramides, seven out of nine species measured (Fig. 2i), and increased the ratio of dihydroceramide to ceramide (Fig. 2j). Inhibiting *de novo* hepatic ceramide biosynthesis may be a mechanism by which FEN can improve insulin sensitivity and glycaemia in mice.

Thus, FEN improved glucose homeostasis without inhibiting obesity or decreasing tissue inflammation in Lepr^*db*^mice. FEN strongly modulated retinoid and metabolic genes in adipose and liver and decreased production of ceramide biosynthesis in liver. FEN also inhibited the elevation in hepatic and adipose *Fgf21* induced by obesity and impaired glucose homeostasis in Lepr^*db*^ mice, demonstrating that the effects of FEN on *Fgf21* gene expression are common to both diet- and genetically-induced obesity.

### Fenretinide treatment inhibits hepatic *Fgf21* expression in Alb-cre-*Ptp1b* ^−/−^ mice, independent of effects on glucose homeostasis

**Figure 3:**
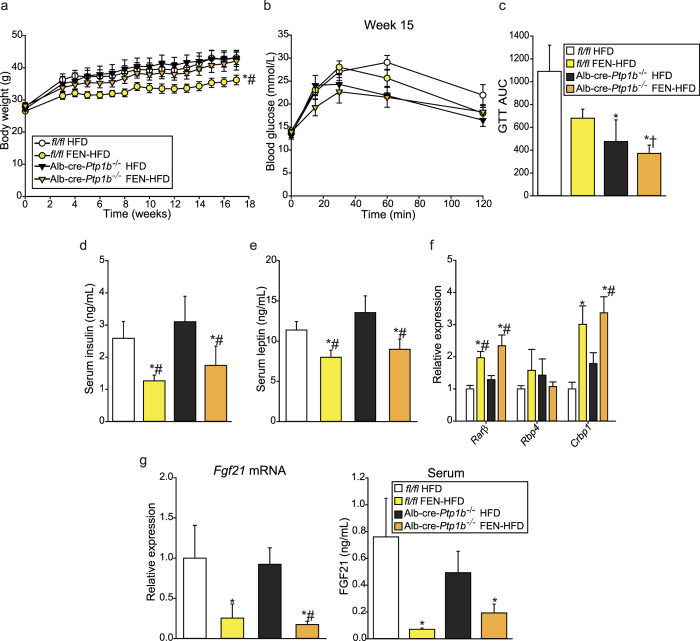
Fenretinide treatment inhibits hepatic *Fgf21* expression in Alb-cre-*Ptp1b*^−/−^ mice, independent of effects on glucose homeostasis. (**a**) Body weight of *fl/fl* male mice on HFD (fl/fl-HFD, n = 9) or HFD +FEN (*fl/fl*-FEN-HFD, n = 9) or hepatocyte-specific-*Ptp1b*^−/−^ male mice on HFD (Alb-cre-*Ptp1b*^−/−^-HFD, n = 5) or FEN-HFD (Alb-cre-*Ptp1b*^−/−^-FEN-HFD, n = 7). (**b**) GTT (1 mg/g glucose) and AUC (**c**) at week 15. (**d**) Serum insulin and (**e**) serum leptin levels at the end of the experiment, following a 5-hour fast. (**f**) Hepatic gene expression of retinoid and metabolic genes, normalised to *Hprt*. (**g**) Hepatic mRNA expression and serum levels of FGF21. Significant differences were determined by two-way ANOVA followed by *post-hoc* tests or student’s t-test for GTT AUC Alb-cre-*Ptp1b*^−/−^-FEN-HFD vs *fl/fl*-HFD; *Rarβ* FEN-HFD (both *fl/fl* and Alb-cre-*Ptp1b*^−/−^) vs *fl/fl-*HFD; *Fgf21* FEN-HFD (both *fl/fl* and Alb-cre-*Ptp1b*^−/−^) vs *fl/fl*-HFD and FEN-HFD vs HFD in Alb-cre-*Ptp1b*^−/−^. Differences are marked *p < 0.05 vs *fl/fl*-HFD, ^#^p < 0.05 vs Alb-cre-*Ptp1b*^−/−^-HFD or † vs *fl/fl*-FEN-HFD.

To test whether directly improving hepatic insulin sensitivity and glucose homeostasis in the background of HFD-induced obesity could lead to a reduction in FGF21 levels, we examined mice with a hepatocyte-specific knock-out of PTP1B. *Ptp1b*^*fl/fl*^ (*fl/fl*) and hepatocyte-specific *Ptp1b*^−/−^ (Alb-cre-*Ptp1b*^−/−^) male mice were fed HFD ± FEN for 17 weeks. As previously reported^26^,^27^, Alb-cre-*Ptp1b*^−/−^ mice showed no difference in body weight compared to *fl/fl* controls. FEN inhibited weight gain and adiposity (data not shown) in many Alb-cre-*Ptp1b*^−/−^ mice but this did not reach statistical significance (Fig. 3a).

Alb-cre-*Ptp1b* knock-out resulted in improved glucose homeostasis in both HFD+/− FEN mice. Alb-cre-*Ptp1b*^−/−^ mice fed HFD appeared to be the most glucose tolerant but this was not statistically significant (Fig. 3b,c). Alb-cre-*Ptp1b* knock-out alone did not alter serum insulin (Fig. 3d) or leptin (Fig. 3e) levels but FEN treatment decreased both factors in each genotype. FEN also increased retinoid gene expression in both *fl/fl* and Alb-cre-*Ptp1b*^−/−^ mice, but there was no effect on hepatic *Rbp4* expression (Fig. 3f). Importantly, *Fgf21* expression and serum levels were similar in Alb-cre-*Ptp1b*^−/−^ and *fl/fl* mice with HFD-induced obesity, but FEN strongly decreased FGF21 in both groups (Fig. 3g). Thus, hepatic *Fgf21* levels are not decreased as a result of improved hepatic insulin signalling alone, thereby providing further evidence for a direct effect of FEN on hepatic *Fgf21*, unrelated to improved insulin sensitivity and glucose homeostasis. However, diverse treatments to improve glycaemic control in humans can decrease elevated levels of FGF21, so it is unclear if there is a direct relationship between glucose homeostasis and FGF21 levels.

### Fenretinide inhibits hepatic *Fgf21* and induces changes to gene expression in lean mice fed a normal-fat diet

**Figure 4:**
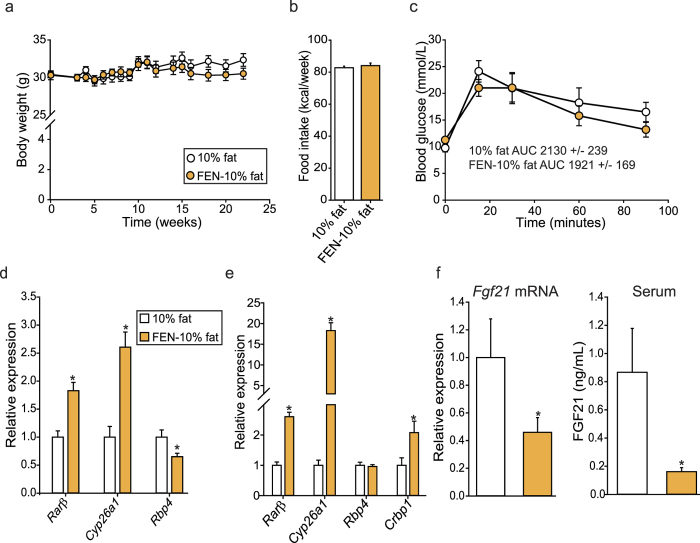
Fenretinide inhibits hepatic *Fgf21* and induces changes to gene expression in lean mice fed a normal-fat diet. (**a**) Body weight of C57BL/6 mice on 10% fat diet (n = 10) or 10% fat +FEN (FEN-10% fat, n = 10). (**b**) Weekly food intake of mice during weeks 2–7. (**c**) GTT (2 mg/g body weight) at week 18. Student’s t-test was used to determine significant differences (none). Expression of retinoid target genes was measured in PG-WAT (**d**) and liver (**e**). Gene expression was normalized to the geomean of *18s, NoNo* and *yWhaz* (PG-WAT) or geomean of *NoNo, yWhaz* and *β-actin* (liver). (**f**) hepatic gene expression and serum levels of FGF21. Significant differences were determined by student’s t-test. Differences are marked *p < 0.05 vs control.

Next, we tested whether FEN could decrease body weight, improve glucose homeostasis and decrease FGF21 levels in lean mice fed a normal-fat diet. FEN had no effect on body weight, food intake (Fig. 4a,b) or glucose tolerance (Fig. 4c) but altered retinoid gene expression in PG-WAT, with increased *Rarβ* and *Cyp26a1* and decreased *Rbp4* expression (Fig. 4d). FEN also increased hepatic *Rarβ, Cyp26a1* and *Crbp1* (Fig. 4e). Strikingly, FEN decreased hepatic *Fgf21* and circulating protein levels in these mice (Fig. 4f). These results suggest that FEN can inhibit basal hepatic *Fgf21* expression but cannot decrease body weight or improve glucose homeostasis under normal physiological conditions.

### Mice with reduced RA synthesis have increased *Fgf21* expression

**Figure 5:**
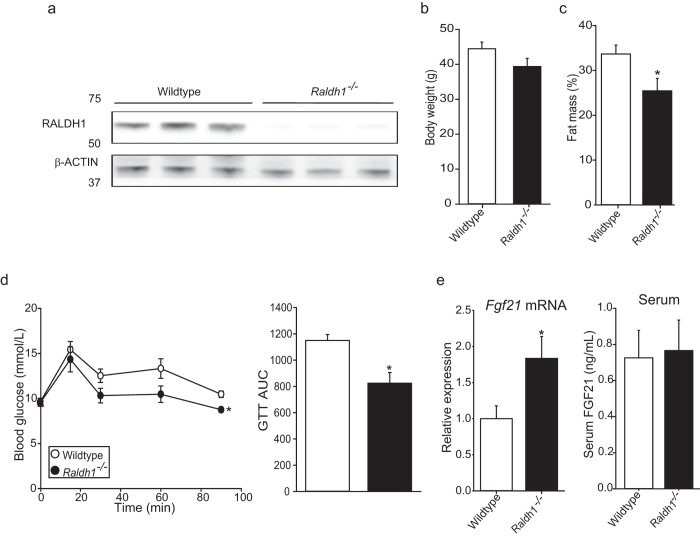
Mice with reduced RA synthesis have increased *Fgf21* expression. (**a**) Representative immunoblot of hepatic RALDH1 levels from C57BL/6 wild type (n = 7 for whole group) and C57BL/6-*Raldh1*^−/−^ mice (n = 8 for whole group). (**b**) Body weight and fat mass (**c**) of all mice. (**d**) GTT (1 mg/g glucose) and AUC analysis at week 10. (**e**) Hepatic *Fgf21* expression (normalized to *Hprt*) and FGF21 serum levels. Significant differences were determined by student’s t-test. Differences are marked *p < 0.05 vs wildtype.

To establish whether our findings were retinoid dependent, we used mice lacking the retinol metabolising enzyme retinaldehyde dehydrogenase 1 (*Raldh1*^−/−^; liver western blot Fig. 5a shows deletion), which have an approximately 70% reduction in normal RA levels^28^. *Raldh1*^−/−^ mice showed decreased fat mass (Fig. 5b,c) and improved glucose tolerance (Fig. 5d) compared to controls. *Raldh1*^−/−^ mice showed increased hepatic gene expression of *Fgf21* but not circulating levels (Fig. 5e). These data suggest that *Fgf21* may be directly suppressed by retinoid-dependent signalling mechanisms.

### Acute retinoid treatment results in inhibition of hepatic *Fgf21* expression and RARα binding at the *Fgf21* promoter is reduced with Fenretinide treatment

**Figure 6:**
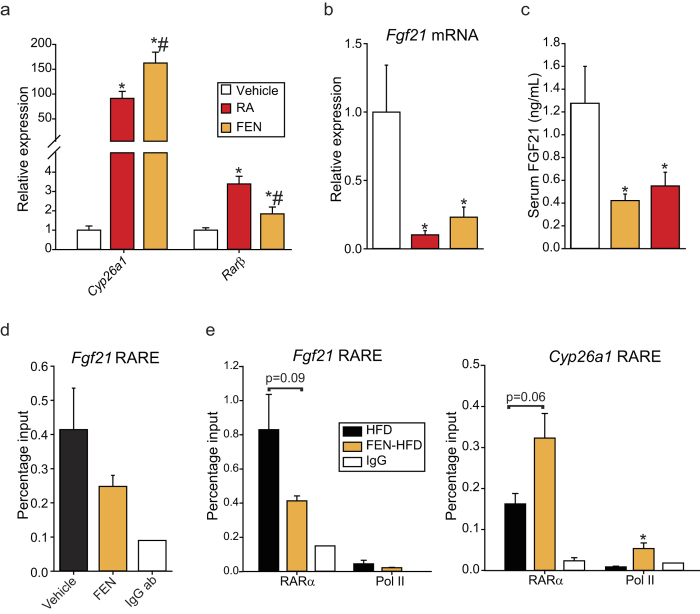
Acute retinoid treatment results in inhibition of hepatic *Fgf21* expression and RARα binding at the *Fgf21* promoter is reduced with Fenretinide treatment. C57BL/6 mice were on chow diet and *ad libitum* fed before a single injection of olive oil vehicle or 50 mg/kg RA or FEN for 2 hours, n = 8 for each group. (**a**) Hepatic gene expression of retinoid targets normalised to *yWhaz*. (**b**) Hepatic *Fgf21* expression and (**c**) serum FGF21 levels. Significant differences were determined by one-way ANOVA followed by *post-hoc* tests or student’s t-test for *Cyp26a1* FEN vs RA, *Rarβ* FEN vs vehicle, *Fgf21* mRNA FEN vs vehicle and FGF21 serum FEN vs vehicle. Differences are marked *p < 0.05 vs vehicle or ^#^p < 0.05 vs RA. (**d**) ChIP-qPCR of RARα binding at the hepatic *Fgf21* RARE in mice injected with vehicle or 50 mg/kg FEN for 2 hours (n = 2 IP’s from 3 individual mice per group). IgG antibody was used to measure non-specific binding (IgG ab, n = 1). (**e**) ChIP-qPCR of RARα and polymerase (Pol) II binding at the hepatic *Fgf21* and *Cyp26a1* RAREs in mice fed HFD or HFD +FEN (FEN-HFD) for 20 weeks (n = 1 IP from 4 individual mice per group). IgG antibody was used to measure non-specific binding (IgG ab). Significant differences were determined by student’s t-test. Differences are marked *p < 0.05.

Since FEN is an activator of both retinoid- and non-retinoid signalling pathways, we tested whether *Fgf21* could be directly inhibited by RA. RA and FEN both increased hepatic target genes *Cyp26a1* and *Rarβ* in lean C57BL/6 mice as expected (Fig. 6a) and in parallel decreased hepatic gene expression and circulating levels of FGF21 at two hours post-injection (Fig. 6b,c).

A putative retinoic acid response element (RARE) was previously reported within the *Fgf21* promoter region^29^. We confirmed the presence of this RARE by ChIP-qPCR and observed that 2-hour FEN treatment resulted in decreased RARα binding at this site (Fig. 6d). Furthermore, both RARα and polymerase (Pol) II binding were decreased at the *Fgf21* RARE in mice chronically fed FEN-HFD, compared to HFD-fed mice (Fig. 6e). Binding of both RARα and Pol-II were increased at RARE 1 of *Cyp26a1* (Fig. 6e), which is induced by retinoid signaling. Together, these results suggest that retinoid signalling has a direct inhibitory effect upon *Fgf21* gene expression.

To test whether acutely blocking RAR signalling could prevent the retinoid-induced suppression of *Fgf21,* we performed experiments in mouse primary hepatocytes. Treatment with GW7647, a potent PPARα agonist, led to the induction of *Fgf21* and *Cd36* (Supplemental Figure S1a). However, RA treatment did not lead to the induction of *Rarβ* or the suppression of *Fgf21* in these cells (Supplemental Figure S1a). Strikingly, *Rarα* gene expression was found to be more than 10-fold lower in mouse primary hepatocytes than in mouse liver tissue (Supplemental Figure S1b). Thus, we suggest that down-regulation of RAR-signalling can prevent the retinoid-induced suppression of *Fgf21*.

## Discussion

FGF21 is an important regulator of lipid and glucose metabolism and is undergoing trials in humans to evaluate its efficacy as a treatment for obesity and type-2 diabetes^3^. However, circulating levels of FGF21 are elevated in obesity and insulin-resistant states, therefore it is not clear if attempts to (in)directly increase levels further will be of therapeutic benefit in humans^8^,^11^,^14^. We have now identified FGF21 as a novel retinoid-dependent target of FEN and thus found a novel direct way to normalise elevated levels in both diet- and genetically-induced obese states whilst, at the same time, improving metabolic dysfunction.

Many others have reported that diverse treatments to improve glycaemic control in humans can decrease elevated levels of FGF21, e.g. with metformin, bariatric surgery or exercise^8^,^9^,^12^,^14^. Although the mechanism by which FGF21 is physiologically induced by fasting, protein restriction and sugar intake is relatively clear, the molecular pathway by which the paradoxical elevation of FGF21 occurs in metabolically dysregulated states is still unknown. We have shown that HFD-feeding itself does not directly induce hepatic FGF21, suggesting that chronic dysregulation of lipid and glucose homeostasis is responsible for the elevation in obese-insulin-resistant states. Moreover, improving hepatic insulin sensitivity and glucose homeostasis in the background of HFD-induced obesity did not decrease FGF21 levels in mice lacking hepatic PTP1B. Thus, more research is required to fully understand the nutritional and pharmacological regulation of FGF21 in physiological and patho-physiological states.

There have been some studies into the negative regulation of FGF21 e.g. PGC-1α potentiates the repressive activity of Rev-Erbα in concert with recruitment of the corepressors NCoR2 and HDAC3 on the FGF21 promoter in primary hepatocytes^24^. Our data, that RARα binding is reduced by FEN treatment at the *Fgf21* promoter, suggests that *Fgf21* is also repressed by RAR-mediated signaling *in vivo*. LXR is also reported to inhibit FGF21 via recruitment of HDAC3^25^. It is therefore possible that RARα may function via a similar mechanism to inhibit *Fgf21* expression. FEN treatment did not change the expression of Rev-Erbα or LXR target genes (Table 1).

Results from cultured cells have not provided clear evidence for a direct mechanism of retinoid-induced suppression of *Fgf21. Fgf21* was shown to be induced by RARβ signaling in human HepG2 cells^29^. We found that down-regulation of *Rarα* in mouse primary hepatocytes prevented the retinoid-induced suppression of *Fgf21*. It is likely that these irreconcilable results are due to the molecular and physiological differences between cultured cells and *in vivo* systems. Directly activating/blocking RAR signalling in mice via the portal circulation could be a more suitable method to test these findings in the future.

We and others have recently identified FEN as a potential safe treatment for diet-induced obesity and type-2 diabetes in mice^17^,^18^,^19^,^22^. FEN can directly inhibit leptin gene transcription but does not have measureable effects on food intake or energy expenditure^17^,^18^. Here, we have established that FEN does not inhibit excess weight gain driven by hyperphagia and low energy expenditure due to disrupted leptin signalling in Lepr^*db*^ mice. These findings suggest that FEN may interact with leptin signalling to inhibit adiposity, but it is unlikely that a direct link can be determined given the difference in potency of effects between FEN and leptin on these energy balance parameters.

In contrast to the lack of an anti-obesity effect in Lepr^*db*^ mice, FEN decreased fasting hyperglycaemia and glucose intolerance and increased pancreatic islet mass and circulating insulin. However, this result contrasts with our studies in HFD-obese mice where FEN decreased serum insulin in parallel with beneficial effects on adiposity and glucose homeostasis^18^ and to the results shown here in Alb-cre *Ptp1b*^−/−^ HFD-fed mice, where FEN treatment also resulted in decreased serum insulin. These results suggest that with the genetic loss of leptin-signalling, FEN may improve insulin sensitivity via a direct effect on the pancreas, perhaps mediated by RA signalling. Normally, leptin inhibits insulin secretion and biosynthesis to adapt glucose homeostasis to the amount of body fat. However, in obesity, a state of leptin resistance contributes to dysregulation of the adipo-insular axis and promotes the development of hyperinsulinaemia^30^. Moreover, RA can induce pancreatic development and affect the function of pancreatic β-cells, thus RA signalling may be involved in complex cross-talk with insulin and leptin signalling in normal physiology and this may be impaired in pathophysiological states^31^.

However, in the more physiological model of HFD-induced obesity, the mechanism of FEN action to improve glucose homeostasis is more likely due to an effect on adipose tissue to normalise impaired mitochondrial fatty acid beta-oxidation and tricarboxylic acid flux^22^. We and others have already shown that FEN can inhibit the final step in ceramide synthesis through blockade of the enzyme dihydroceramide desaturase 1 in adipose tissue^20^,^22^. Here, we have now shown that ceramide synthesis is also blocked by FEN in liver in a genetic model of severe obesity and insulin resistance. Excess levels of ceramides directly mediate impaired insulin signalling (via inhibition of Akt phosphorylation and dysregulation of mitochondrial fatty acid oxidation^32^,^33^). Thus, FEN may improve glucose homeostasis in mice via decreased ceramide accumulation and normalisation of metabolic flux in adipose, liver and muscle, and/or metabolic flux between these insulin-sensitive tissues, independent of effects on the regulation of FGF21. Further data analysis of our RNA-seq data may reveal the pathways that are altered in association with the beneficial effects of FEN-treatment.

Overall, the list of effects upon FEN treatment now includes suppressing the elevation of FGF21 in insulin resistant states. Since the pleotropic effects of FEN are all beneficial and not at all detrimental, our findings suggest targeting the multiple molecular defects that are manifest with metabolic disease may be an achievable goal.

## Methods

### Animals

Experiments were approved by the University of Aberdeen Ethics Board and performed following UK Home Office licenses PPL60/3951 and PPL70/7911 under the Animals (Scientific Procedures) Act 1986. Male C57BL/6 mice fed HFD ± 0.04% FEN were previously reported (Supplementary Table S1)^18^,^22^. HFD used in respective mouse cohorts through-out this study contained 45% lard-based fat. Male C57BL/6 Lepr^*db*^ and lean wild-type/heterozygous littermates were from Charles River (Edinburgh, UK). Mice were group-housed (2–3 obese/8 lean mice per cage) with conditions as previously reported^18^. Male C57BL/6 *Raldh1*^−/−^ and wild-type mice were bred in-house and described previously^18^. *Ptp1b*^*fl/fl*^ and albumin-cre-*Ptp1b*^−/−^ mice with hepatocyte-specific deletion of protein tyrosine phosphatase (PTP)-1B

(Alb-cre-*Ptp1b*^−/−^) were previously generated^26^,^27^. Alb-cre-*Ptp1b*^−/−^ mice were fed HFD ± FEN as previously reported for C57BL/6 mice^18^,^22^. In FEN-diet studies, both Lepr^*db*^ and C57BL/6 mice were fed 10% fat diet (D12450B, Research Diets, New Brunswick, NJ). Lean controls, C57BL/6 mice for injection studies and *Raldh1*^−/−^ mice were fed chow diet^18^. Glucose tolerance test (GTT) and fat mass measurements by Echo MRI or DEXA scan were performed as previously described^5^,^18^. GTT area under the curve was calculated using time zero glucose level for each mouse. Mice were killed following a 5 h fast (except for injection studies), tissues rapidly dissected, flash frozen in liquid nitrogen and stored at −80 °C until further use.

### Tissue and serum analysis

Tissue serum factors, liver triacylglycerols and gene expression were measured as previously described^5^. Most stable reference genes were selected by screening a panel of common reference genes with RefFinder^34^. Mouse primer sequences used for qPCR are listed in Supplementary Table S2. Pancreas tissues for IHC were rapidly dissected, fixed in formalin and embedded in paraffin wax using a Peloris II tissue processor (Leica Biosystems, Milton Keynes, UK). Embedded tissue was cut into 4 μM-thick sections and mounted onto glass slides. Tissue sections were dewaxed, stained with haematoxylin and eosin, and visualized using an Axioskop 40 microscope (Zeiss, Cambridge, UK). Islet area size was quantified using ImageJ software (NIH, Bethesda, MD, USA).

### RNA sequencing

Total RNA was extracted from frozen liver using TriZOL reagent (Sigma Aldrich, Irvine, UK) to manufacturer’s instructions. RNA was purified using RNeasy miniprep kit (Qiagen, Manchester, UK) and quantified on a Bioanalyzer 2000 (Agilent Technologies, Edinburgh, UK). Ribosomal RNA was removed from samples using the Ribo-Zero rRNA removal kit (Illumina, San Diego, CA, USA). Sequencing libraries were prepared using the TruSeq RNA Library Preparation Kit v2 (Illumina) following the low sample protocol, using 1 μg RNA. Libraries were sequenced on the NextSeq-500 Desktop sequencer platform (Illumina) with a 2 × 75 paired-end read length giving 719 million raw reads (average depth 45 million reads per sample). Reads were filtered using the FASTQ Toolkit v2.0.0 in the Illumina BaseSpace cloud computing environment to remove adapter sequences and reads with phred score <20 or length <20 bp^35^. Filtered reads were checked using FastQC before aligning with STAR 2.0 to the *Mus musculus* UCSC mm10 genome and differential expression measured using DESeq2 with default parameters^36^,^37^,^38^,^39^,^40^.

### Quantification of liver dihydroceramides and ceramides

Extraction of liver lipids was performed according to the method described by Folch *et al*.^41^. Ceramides and dihydroceramides were isolated by solid phase extraction chromatography using C17:0 ceramide and C12:0 dihydroceramide (Avanti Polar Lipids, Alabaster, Al, USA) as internal standards. Samples were analysed by liquidchromatography–mass spectrometry (LC–MS) using a Thermo TSQ Quantum Ultra mass spectrometer (Thermo Scientific, Hemel Hempstead, UK), equipped with a heated electrospray ionisation (HESI) probe and coupled to a Thermo Accela 1250 UHPLC system. Samples were analysed in positive ion mode over the mass to charge (m/z) range 200–2000. The samples were injected on to a Kinetex C8 column (2.1 mm_100 mm, 2.6 μm). Mobile phase A consisted of 90% (v/v) H_2_O_2_ and 10% (v/v) acetonitrile with 0.1% (v/v) formic acid. Mobile phase B consisted of 90:10 isopropanol/acetonitrile with 0.1% (v/v) formic acid. The initial conditions for analysis were 80% B and the percentage of mobile phase B was increased to 100% over 15 minutes and held for 1 min before re-equilibration with the starting conditions for 4 min. All solvents were LC–MS grade (Fisher Scientific, Loughborough, UK). The raw LC–MS data were processed with Thermo Xcalibur v2.1 (Thermo Scientific) and concentration of ceramide and dihydroceramide molecular species determined by comparison to calibration curves generated with C16:0 and C24:1 standards (Avanti). Total ceramide and dihydroceramide concentrations were calculated from the summed concentrations of all the monitored molecular species. All values were normalised to the wet weight of liver.

### Immunoblotting and Chromatin Immunoprecipitation (ChIP)

Tissue lysates were prepared and immunoblotting performed as described previously^42^,^43^. ALDH1A1 (RALDH1; Abcam; ab52492) and β-actin (Pierce Scientific; PA1-21176) primary antibodies were at 1/1000 dilution; peroxidase-conjugated goat-anti-rabbit secondary antibody (Anaspec; 28177) at 1/5000 dilution. ChIP was performed using the SimpleChIP kit (9002, Cell Signalling, Danvers, MA) with antibodies: RARα (C15310155, Diagenode) and Pol II (sc-3001, Santa Cruz).

### Statistical analysis

Sequencing data was analysed with DESeq2 and presented as p values normalised for a false discovery rate of 0.05. All other data was analysed using Sigmaplot 13.0 and presented as mean ± SEM and compared using two-way or one-way ANOVA with *post-hoc* tests or student’s t-test as indicated.

## Additional Information

**How to cite this article:** Morrice, N. *et al*. Elevated Fibroblast growth factor 21 (FGF21) in obese, insulin resistant states is normalised by the synthetic retinoid Fenretinide in mice. *Sci. Rep.* **7**, 43782; doi: 10.1038/srep43782 (2017).

**Publisher's note:** Springer Nature remains neutral with regard to jurisdictional claims in published maps and institutional affiliations.

## Supplementary Material

Supplementary Figures, Tables and Methods

## Figures and Tables

**Table 1:** Hepatic expression of genes regulated by transcription factors that also regulate *Fgf21*.

Gene	Log_2_ fold change	adj. p value	q value
Transcription factors			
* Mlxipl* (ChREBP)	+0.123	0.301	1.000
* Nr1h3* (LXRα)	−0.095	0.431	1.000
* Nr1c1* (PPARα)	+0.040	0.786	1.000
* Nr1c3* (PPARγ)	+0.419	1.000	1.000
* Nr1d1* (Rev-erbα)	−0.261	0.254	1.000
ChREBP targets			
* Gck*	−0.378	0.046	0.894
* Pck1*	+0.410	1.000	1.000
* Elovl6*	−0.288	1.000	1.000
LXR targets			
* Apoe*	−0.211	0.198	1.000
* Fas*	−0.072	0.648	1.000
* Cyp7a1*	−0.196	1.000	1.000
PPARα/PPARγ targets			
* Acot1*	+0.448	1.000	1.000
* Ehhadh*	+0.211	0.786	0.163
* Vnn1*	+0.496	0.094	0.094
* Cidec*	+0.159	1.000	1.000
* Cd36*	−0.211	0.150	1.000
* Mogat1*	+0.088	0.723	1.000
Rev-erbα targets			
* Arntl* (BMAL1)	+0.060	0.786	1.000
* Per1*	−0.021	0.585	1.000
* Cry1*	+0.104	1.000	1.000

DESeq2 analysis of liver RNA-seq of expression of transcription factors known to regulate *Fgf21* and some of their key target genes in mice fed HFD (n = 4) vs HFD +0.04% FEN (FEN-HFD, n = 4) for 7 days.

**Table 2:** Gene expression, serum factors and metabolites in Lepr^*db*^ mice.

Gene	Lean	Lepr^*db*^	FEN-Lepr^*db*^
Adipose: metabolic genes			
* C/ebpα*	1.00 ± 0.13	0.52 ± 0.05*	0.89 ± 0.19
* Chrebp1*	1.00 ± 0.14	0.40 ± 0.07*	0.65 ± 0.10*
* Fas*	1.00 ± 0.23	0.26 ± 0.03*	0.58 ± 0.14^#^
* Glut4*	1.00 ± 0.13	0.36 ± 0.06*	0.63 ± 0.14
* Insulin receptor*	1.00 ± 0.12	0.32 ± 0.04*	0.41 ± 0.08*
* Pparγ*	1.00 ± 0.14	0.52 ± 0.09*	0.83 ± 0.19
* Scd1*	1.00 ± 0.14	0.55 ± 0.06*	0.69 ± 0.06*
Adipose: retinoid genes			
* Crbp1*	1.00 ± 0.28	0.81 ± 0.17	1.07 ± 0.37
* Raldh1*	1.00 ± 0.26	0.54 ± 0.13	0.65 ± 0.18
* Rarγ*	1.00 ± 0.16	0.37 ± 0.09*	0.56 ± 0.11*
* Rbp4*	1.00 ± 0.38	3.61 ± 0.99*	0.88 ± 0.46^#^
Adipose: adipokine genes			
* Adiponectin*	1.00 ± 0.18	0.22 ± 0.06*	0.21 ± 0.03*
* Leptin*	1.00 ± 0.24	2.30 ± 0.83	1.49 ± 0.39
* Resistin*	1.00 ± 0.18	0.19 ± 0.06*	0.11 ± 0.02*
Adipose: inflammation genes			
* Cd11b*	1.00 ± 0.15	3.34 ± 0.95*	2.98 ± 0.54*
* F4/80*	1.00 ± 0.32	24.47 ± 5.66*	22.91 ± 6.16*
Liver: retinoid genes			
* Crbp1*	1.00 ± 0.15	1.75 ± 0.27*	22.26 ± 10.98*^,#^
* Cyp26a1*	1.00 ± 0.11	0.31 ± 0.07*	47.77 ± 3.13*^,#^
* Lrat*	1.00 ± 0.09	2.70 ± 0.29*	3.41 ± 0.36*
* Rbp4*	1.00 ± 0.09	1.98 ± 0.20*	1.30 ± 0.13*^,#^
* Rarβ*	1.00 ± 0.14	3.19 ± 0.47*	8.79 ± 1.02*^,#^
Liver: inflammation genes			
* Cd11b*	1.00 ± 0.25	3.12 ± 1.32	3.32 ± 0.41*
* F4/80*	1.00 ± 0.16	4.94 ± 1.78*	3.10 ± 1.05*
Hypothalamus genes			
* Foxo1a*	1.00 ± 0.18	0.43 ± 0.04*	0.52 ± 0.13*
* Melanocortin 4 receptor*	1.00 ± 0.26	0.49 ± 0.06	0.37 ± 0.03*
* Npy*	1.00 ± 0.15	1.61 ± 0.32*	1.85 ± 0.39
* Npy receptor*	1.00 ± 0.22	0.62 ± 0.07	0.44 ± 0.05*
* Pomc*	1.00 ± 0.12	0.16 ± 0.06*	0.22 ± 0.04*
* Ptp1b*	1.00 ± 0.13	0.48 ± 0.06*	0.49 ± 0.06*
* Raldh2*	1.00 ± 0.13	0.76 ± 0.08*	0.96 ± 0.21
* Stat3*	1.00 ± 0.20	0.49 ± 0.04*	0.46 ± 0.08*
* Tgfβ1*	1.00 ± 0.26	0.54 ± 0.07	0.56 ± 0.09
Serum leptin	5.60 ± 1.63	131.48 ± 3.34*	134.88 ± 6.09*
Liver triglycerides	8.34 ± 0.89	53.75 ± 5.40*	61.97 ± 9.73*

Relative mRNA in PG-WAT, liver and hypothalamus and serum factors and metabolites in lean or Lepr^*db*^ mice+/−FEN. Expression was normalised to *Nono* in liver or *yWhaz* in adipose and hypothalamus. Data are shown as mean ± SEM and significance determined by one-way ANOVA followed by *post-hoc* tests or student’s t-test for liver: *Crbp1* Lepr^*db*^ vs lean, *Rbp4* FEN-Lepr^*db*^ vs lean*, Cd11b* FEN-Lepr^*db*^ vs lean and *F4/80* FEN-Lepr^*db*^ vs lean and Lepr^*db*^ vs lean groups; PG-WAT: *Scd1* Lepr^*db*^ vs lean, *Fas* FEN-Lepr^*db*^ vs Lepr^*db*^, *Pparγ* Lepr^*db*^ vs lean, *C/ebpα* Lepr^*db*^ vs lean, *Fgf21* Lepr^*db*^ vs lean, *Rarγ* FEN-Lepr^*db*^ vs lean, *Cd11b* Lepr^*db*^ vs lean, *Fgf21* FEN-Lepr^*db*^ vs Lepr^*db*^ and Lepr^*db*^ vs lean and *β-klotho* FEN-Lepr^*db*^ vs lean and Lepr^*db*^ vs lean groups; hypothalamus: *Npy* Lepr^*db*^ vs lean, and *Raldh2* Lepr^*db*^ vs lean groups. Differences are marked *p < 0.05 vs lean; ^#^p < 0.05 vs Lepr^*db*^.
