# Supplementary material for: Elevated Fibroblast growth factor 21 (FGF21) in obese, insulin resistant states is normalised by the synthetic retinoid Fenretinide in mice
Source: Sci Rep. 2017 Mar 3;7:43782. doi: 10.1038/srep43782 (PMC5335663; doi:10.1038/srep43782)
Supplement: Supplementary Figures, Tables and Methods [file srep43782-s1.pdf]

Supplementary data and methods for:

Elevated Fibroblast growth factor 21 (FGF21) in obese, insulin resistant states is normalised by the synthetic retinoid Fenretinide in mice

Nicola Morrice[1,2], George D. Mcilroy[1], Seshu R. Tammireddy[3], Jennifer Reekie[1], Kirsty D. Shearer[1], Mary K. Doherty[3], Mirela Delibegovic[1], Phillip D. Whitfield[3] and Nimesh Mody[1]*

1. Institute of Medical Sciences, College of Life Sciences and Medicine, University of Aberdeen, Foresterhill Health Campus, Aberdeen, Scotland, UK, AB25 2ZD

2. Centre for Genome Enabled Biology and Medicine, University of Aberdeen, 23 St Machar Drive, Old Aberdeen, Aberdeen, Scotland, UK, AB24 3UU

3. Lipidomics Research Facility, Department of Diabetes and Cardiovascular Science, University of Highlands and Islands, Old Perth Road, Inverness, Scotland, IV2 3JH

* Corresponding author: Nimesh Mody, tel: +44 (1224) 437102; email: n.mody@abdn.ac.uk

Supplementary table S1

Measurement	Chow	HFD	FEN-HFD
**20 weeks diet**			
Final body weight (g)	31.8 ± 0.6	45.2 ± 0.87 *	39.8 ± 1.5 *#
Adiposity at 12 weeks (fat mass in g)	4.6 ± 0.2	13.4 ± 0.5 *	10.3 ± 0.9 *#
Serum glucose at 12 weeks (mmol/L)	11.9 ± 0.7	20.9 ± 0.2 *	11.6 ± 0.6 #
Serum insulin at 20 weeks (ng/mL)	0.7 ± 0.01	2.8 ± 0.3 *	1.6 ± 0.2 *
Serum leptin at 20 weeks	1.0 ± 0.3	1.5 ± 0.3 *	1.6 ± 0.2 *#
Food intake from chronic cumulative study (kcal/ day)	-	14.2 ± 0.2	13.8 ± 0.3
**7 days diet**			
Final body weight (g)	24.7 ± 0.7	26.2 ± 0.2	25.6 ± 0.3
Serum leptin	1.0 ± 0.2	4.6 ± 0.8 *	2.4 ± 0.5 #
Food intake from acute study (average change in energy intake from baseline in kcal/day)	- 0.3 ± 0.2	2.2 ± 0.8 *	1.8 .± 0.4 *

Supplementary table S1: Metabolic parameters measured in mice fed HFD for 7 days or 20 weeks

Data was previously published in Mcilroy GD et al, Fenretinide Treatment Prevents Diet-Induced Obesity in Association With Major Alterations in Retinoid Homeostatic Gene Expression in Adipose, Liver and Hypothalamus, *Diabetes*, 2013 Mar; 62(3): 825- 836.

Data are shown as mean ± SEM and significance was determined by one-way ANOVA followed by *post-hoc* tests. Differences are marked * p<0.05 vs chow; #p<0.05 vs FEN-HFD.

Supplementary figure S1

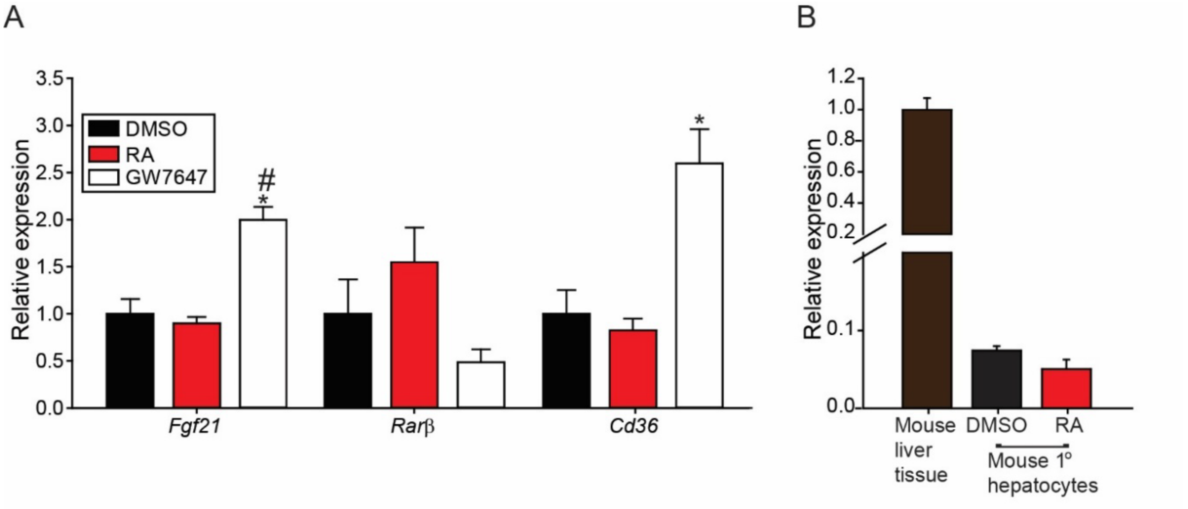

Supplementary figure S1: Downregulation of retinoid signalling in mouse primary hepatocytes prevents the inhibition of *Fgf21* expression by RA

A: qPCR analysis of gene expression in mouse primary hepatocytes treated overnight with DMSO followed by DMSO (black bars) or 1µM RA (red bars) the following day for 60 min or 1µM GW7647 overnight followed by 60 min DMSO treatment (white bars); n = 3 per treatment. Gene expression was normalised to *yWhaz*.

Significant differences were determined by student's t-test. Differences are shown as * $p<0.05$ vs DMSO + DMSO; # $p<0.05$ vs DMSO + RA.

B: Semi-quantitative analysis of Rarα gene expression in mouse primary hepatocytes treated overnight with DMSO followed by DMSO (black bar) or 1µM RA (red bar) the for 60 min (gene expression for both normalised to *yWhaz*), compared to gene expression levels in liver from chow-fed lean control mice (n=10; gene expression normalised to *Nono*).

Supplementary table S2

Gene	Forward	Reverse
18s	CGTTCCTTACCTGGTTGAT	GAGCGACCAAAGGAACCATTA
Adiponectin	TGTTCCTCTTAATCCTGCCCA	CCAACCTGCACAAGTTCCCTT
Beta-actin	GATCTGGCACCACACACCTTC	GGGGTGTTGAAGGTCTCAAA
Beta- klotho	TGTTCTGCTGCGAGCTGTTAC	CCGGACTCACGTACTGTTTTT
C/ebp-alpha	CAAGAACAGCAACGAGTACCG	GTCACTGGTCAACTCCAGCAC
Cd11b	CCCCACACTAGCATCAAGGG	GAGGCAAGGGACACATGAC
Cd36	GAACCACTGCTTTCAAAAACTGG	TGCTGTTCTTTGCCACGTCA
Chrebp1	AGATGGAGAACCGACGTATCA	ACTGAGCGTGCTGACAAGTC
Crbp1	CTGAGCAATGAGAATTTCGAGGA	GCGGTCGTCTATGCCTGTC
Cyp26a1	TTCGGGTTGCTCTGAAGACT	TCCTCCAAATGGAATGAAGC
Cyp26a1 RARE	CCGAATTAAAGATGAACTTTG	GCACGCTTCAGCCTCCCGCGCC
F4/80	CCCAGCTTATGCCACCTGCA	TCCAGGCCCTGGAACATTGG
Fas	AAGCTCAGTGTGCCCACCTA	ATGGCAACGTGACACTGCTG
Fgf21	ACCTGGAGATCAGGGAGGAT	CACCCAGGATTTGAATGACC
Fgf21 RARE	CCTCAGACCCAAGAGCCAGA	AAGGAGGAGGCTGGGGTCTA
Fgfr1	GGTGAACGGGAGTAAGATCGG	CCCCGCATCCTCAAAGGAG
Glut 4	GTGACTGGAACACTGGTCCTA	CCAGCCACGTTGCATTGTAG
Hprt	GTTAAGCAGTACAGCCCCAAA	AGGGCATATCCAACAACAAACTT
Insulin receptor	AGACCAACTGTCCTGCCACT	ACACACTTGGTGGGGTCATC
Leptin	GAGACCCCTGTGTCGGTTC	CTGCGTGTGTGAAATGTCATTG
Lrat	CCGTCCCTATGAAATCAGCTC	ATGGGCGACACGGTTTTTCC
Mc4r	CCCGGACGGAGGATGCTAT	TCGCCACGATCACTAGAATGT
NoNo	GCCAGAATGAAGGCTTGACTAT	TATCAGGGGGAAGATTGCCCA
Npy	ATGCTAGGTAACAAGCGAATGG	TGTCGCAGAGCGGAGTAGTAT
Pepck	GAGATAGCGGCACAAT	TTCAGAGACTATGCGGTG
Pomc	GTGCCAGGACCTCACCACGG	CGTTGCCAGGAAACACGGGC
Pparα	ACGATGCTGTCCTCCTTGATG	GTGTGATAAAGCCATTGCCGT
Pparγ	AGTGGAGACCGCCCAGG	GCAGCAGGTTGTCTTGGATGT
Ptp1b	GGAACTGGGCGGCTATTTACC	CAAAAGGGCTGACATCTCGGT
Rarβ	CGCGAGCCCTTCCTCCTGC	AAAAGCCCTTGCACCCCTCGC
Rarγ	GGAGCAGGCTTCCCATTCG	CATGGCTTATAGACCCGAGGA
Raldh1	GGTGGAGGACGCTGGGGGAA	CCGAAGGGGCACTGGGCTGA
Raldh2	CAAGGAGGCTGGCTTTCCACCC	GGGCTCTTCCCTCCGAGTTCCA
Rbp4	ACGAGTCCGTCTTCTGAGCAACTG	GCACAGCTCCTCCTGCCGTT
Resistin	AAGAACCTTTCATTTCCCCTCCT	GTCCAGCAATTTAAGCCAATGTT
Scd1	TTCTTGCGATACACTCTGGTGC	CGGGATTGAATGTTCTTGTCGT
Stat3	CAATACCATTGACCTGCCGAT	GAGCGACTCAAACTGCCCT
Tgfβ1	AGCCCGAAGCGGACTACTAT	CTGTGTGAGATGTCTTTGGTTTTC
yWhaz	GAAAAGTTCTTGATCCCCAATGC	TGTGACTGGTCCACAATTCCTT

Supplementary table S2: Mouse primer sequences used in qPCR reactions.

Supplementary methods

Mouse primary hepatocyte cell culture

Mouse primary hepatocytes were extracted from a C57BL/6 wild-type mouse using a modified version of the collagenase method[1]. Tissue was perfused via the inferior vena cava with 137mM NaCl, 7mM KCl, 0.7mM Na_2HPO_4, 10mM Hepes, 0.5mM EDTA, pH 7.65 (perfusion buffer) followed by 137mM NaCl, 7mM KCl, 0.7mM Na_2HPO_4, 10mM Hepes, pH 7.65 (wash buffer) and finally with perfusion buffer plus added 5.1mM $CaCl_2$ and 20mg collagenase (from *C.histolyticum*). All perfusions were performed at 5ml/min flow rate. Cells were strained and seeded at a density of 2.5 x 10^5 cells/ well (6 well plate) in M199 + glutamax, with added 1% (v/v) Pen-strep, 0.1% (v/v) BSA, 10% (v/v) FCS, 10nM insulin Actrapid, 200nM 3,3',5-triiodo-L-thyronine and 500nM dexamethasone, and incubated at 37°C, 5% CO_2. After 4 hours, media was replenished and supplemented with compounds as per appropriate figure legends. The following day, plating media was replaced with M199+ glutamax with added 1% (v/v) Pen-strep and 100nM dexamethasone, supplemented with compounds as per respective figure legends.

References

1. Berry, M. N. & Friend, D. S. High-yield preparation of isolated rat liver parenchymal cells: a biochemical and fine structural study. *J. Cell Biol.* **43**, 506-520 (1969).
